# Surface Electromyographic (SEMG) Biofeedback for Chronic Low Back Pain

**DOI:** 10.3390/healthcare4020027

**Published:** 2016-05-17

**Authors:** Randy Neblett

**Affiliations:** Productive Rehabilitation Institute of Dallas for Ergonomics (PRIDE) Research Foundation, 5701 Maple Ave. #100, Dallas, TX 75235, USA; randyneblett@pridedallas.com; Tel.: +1-214-351-6600

**Keywords:** surface electromyography (SEMG), biofeedback, flexion-relaxation, chronic low back pain (CLBP)

## Abstract

Biofeedback is a process in which biological information is measured and fed back to a patient and clinician for the purpose of gaining increased awareness and control over physiological domains. Surface electromyography (SEMG), a measure of muscle activity, allows both a patient and clinician to have direct and immediate access to muscle functioning that is not possible with manual palpation or visual observation. SEMG biofeedback can be used to help “down-train” elevated muscle activity or to “up-train” weak, inhibited, or paretic muscles. This article presents a historical and clinical overview of SEMG and its use in chronic low back pain assessment and biofeedback training.

## 1. A Historical Overview of Biofeedback

Biofeedback is a process in which biological information is measured and fed back to a patient and clinician. Though biofeedback technology can be used for diagnostic purposes, it is most often used for self-regulation skills training. The goal of biofeedback training is to teach increased awareness and control over biological process. Due to limitations in the available technology at the time, first generation biofeedback equipment provided only an analogue needle display or a simple sound. As the technology improved in the 1970s, digital numerical displays became available. Biofeedback became computerized in the 1980s with the first Apple computers. Today, biofeedback has become much more sophisticated, allowing computerized multimedia colorful interactive displays and sounds, and allowing detailed recordings and statistical analysis of biological information. Despite these advances, the accuracy and meaningfulness of a biofeedback signal is dependent on the grade of the equipment and the skill of the practitioner, in knowing proper skin preparation, sensor placement, measurement settings, display setup, and signal interpretation [1]. A good source for biofeedback training and certification is the Biofeedback Certification International Alliance [2]. 

A number of biofeedback modalities are used in chronic pain management and physical rehabilitation, including autonomic nervous system measures (hand temperature, skin conductance, and heart rate), central nervous system measures (electroencephalography (EEG)), and biomechanical measures (force and pressure) [3]. Surface electromyography (SEMG) is one of the oldest biofeedback modalities [4]. It was used in clinical research as early as the 1920s, by Edmond Jacobson, the developer of the progressive muscle relaxation therapeutic technique [5,6]. When speaking with patients, clinicians often describe SEMG as measuring "muscle tension," but it is actually a measure of the electrical activity generated by muscle action potentials, which are rapid electrical signals that travel along the surface of the motor end plate, resulting in a muscle contraction [7]. SEMG allows both a patient and a clinician to have direct and immediate access to muscle functioning that is not possible with manual palpation or visual observation. Three electrodes are required to measure a single muscle area: one positive, one negative, and one reference. In biofeedback terminology, this is called a “placement.” When assessing the lumbar muscles, for example, electrodes are most often applied vertically on the erector spinae muscles, with equal distance between the sensors, in two separate placements, on the left and right side of the back [8]. When measuring SEMG with a standard biofeedback instrument, the raw SEMG signal is usually converted to a root mean square, rectified SEMG signal, for easier interpretation [1]. Depending on one’s clinical or research purposes, the practitioner can further modify the measurement characteristics of the signal (such as sampling rates and filters) and display characteristics of the signal (such as gain and smoothing) [1]. SEMG biofeedback has been used for general relaxation training, stroke rehabilitation, and treatment of pain. SEMG can be used to help “down-train” elevated muscle activity or to “up-train” weak, inhibited, or paretic muscles [3].

Traditional biofeedback involves an operant conditioning process in which movement toward the desired goal is shaped over time, with minimal therapist instruction [9]. This training methodology works well with autonomic nervous system modalities, in which the training goal is to lower general autonomic nervous system arousal. In fact, it is likely that most biofeedback practitioners who treat chronic pain patients use SEMG in this way. A very common SEMG placement for general relaxation training is to place one active electrode above each eye on the forehead. This Frontalis placement has historically been seen as a good indicator of emotional distress and an indicator of general tension in the rest of the body, although this second assumption has not been shown to be true [10,11]. In fact, early studies of SEMG biofeedback training for pain treatment tended to focus on the Frontalis muscle, regardless of the painful body part that was being treated [12]. Regardless of these old assumptions, stress is well known to exacerbate pain, and many chronic pain patients have poor coping strategies for managing stress, so any skills that patients can learn to help them relax, including SEMG biofeedback training to lower facial tension or other muscle tension in the body, may be found to be beneficial. General relaxation training with a variety of methods, with or without biofeedback assistance, has been shown to be an effective treatment for pain [13].

In contrast to traditional biofeedback, SEMG biofeedback for purposes of muscle re-education lends itself to a more direct coaching methodology [14]. A number of specific SEMG biofeedback protocols for treating pain have been described in the scientific literature [10,14,15,16,17,18,19,20,21,22,23,24]. Although EMG biofeedback is the most widely used and widely reported method of biofeedback in chronic pain treatment and rehabilitation, few clinical SEMG biofeedback training protocols have been scientifically tested and published in peer-reviewed journals [3]. The dearth of randomized control studies is perhaps due, at least in great part, to the difficulty of designing studies with credible sham SEMG feedback, which can be compared with real SEMG feedback, in assessing the efficacy of a defined biofeedback training protocol.

There are generally two goals with SEMG biofeedback training: to increase awareness of the target muscle(s); and to increase control of the muscle(s). A primary concept regarding SEMG biofeedback training with chronic pain patients is that they tend to be very poor at knowing how tense they are when compared with pain-free controls [22]. A seminal study, with chronic low back pain (CLBP) subjects, found a deficit in their ability to discriminate between higher and lower tension levels and a tendency to underestimate tension levels when they were elevated. This difficulty in estimating tension levels was found in both painful and non-painful muscles sites. It was suggested by the study's authors that these deficits may lead patients to believe that elevated muscle tension levels are in a normal range, which may preclude adjustments to lower tension levels [25]. This type of deficit in muscle awareness has been observed in thousands of chronic pain patients by this author.

## 2. SEMG with Chronic Low Back Pain

Lumbar SEMG measures, in both static and dynamic postures and movements, have been found to be reliable in both normal and CLBP subjects, and both within and between sessions [26,27,28,29,30]. Some studies have found that CLBP patients display lower SEMG levels than controls during certain movements [31,32,33]. In addition, static left/right asymmetries have been reported in the lumbar musculature [34,35]. Despite the commonly held assumption that muscle bracing can cause increased pain, most studies have found no significant relationship between static SEMG levels and subjective pain reports [36,37]. When comparing low back pain subjects *vs.* control subjects in various static postures, the results have been equivocal, with some studies reporting significant differences, and others not [38]. However, EMG biofeedback training of lumbar muscles in static postures has been found to be associated with significant improvements in cognitive and behavioral indices of CLBP for up to a 2.5 year follow-up [39]. Flor *et al*. (1991) found that SEMG biofeedback of the lumbar muscles in subjects with “mild” chronic back pain was superior to cognitive behavioral counseling (including relaxation without biofeedback) and medical “treatment as usual” in outcome measures of pain, functional interference, and affective distress [12]. A similar study, which replicated aspects of the biofeedback portion of the Flor *et al.* (1991) study, found clinical improvements in both the SEMG biofeedback and cognitive behavioral groups, with no significant differences between the two [40]. In a separate study of CLBP subjects with more severe functional limitations, a combination of cognitive interventions and self-regulation skills training (including SEMG biofeedback) was found to be most efficacious in clinical outcome measures [41]. Correcting muscle imbalances in CLBP subjects have been reported to result in decreased pain, with gains lasting up to four years post-treatment [35]. Interestingly, a comparison group, which was educated on lumbar muscle symmetry, demonstrated similar gains in pain to the SEMG biofeedback group. The addition of SEMG biofeedback to a traditional exercise program for CLBP subjects resulted in a significant increase in lumbar strength measures [42]. Whether the strength improvement was due to an actual change in the muscles or to a reduction of fear avoidance could not be determined.

## 3. The Flexion-Relaxation Phenomenon

SEMG as an assessment tool has a long history within low back pain research. Beginning in the early 1950s, researchers first began evaluating lumbar muscle activity in different postures and movements [43,44,45,46]. This is when the flexion-relaxation (FR) phenomenon was first discovered, in which lumbar muscles relax completely at maximum voluntary flexion. The FR phenomenon is perhaps the most studied pattern of lumbar SEMG activity. It has now been found in many studies that this FR pattern can be reliably measured in most normal subjects, but it is often absent in CLBP patients [26,27,28,29,45,47,48,49,50,51]. An extensive meta-analysis of lumbar SEMG found that measures of FR in previous studies have produced a large effect size (*d* = −1.71) in distinguishing between CLBP patients and control subjects [38]. FR deficits in low back pain subjects have also been found to be associated with self-reported disability [52], pain [44,53,54], and fear of pain and re-injury [55]. When assessed as a treatment outcome measure, positive treatment changes in FR with CLBP patients have been found to be associated with clinical improvement in self-efficacy beliefs, fear avoidance beliefs [28], perceived disability, pain intensity, and range-of-motion (ROM) [56].

More recently, attempts have been made to actively modify abnormal FR in CLBP patients with SEMG biofeedback training. A biofeedback training protocol of surface EMG-assisted stretching (SEMGAS) has been described, which teaches CLBP patients how to relax into standing maximum voluntary flexion and achieve FR [57,58,59]. This procedure was first introduced in the context of an interdisciplinary functional restoration program. The first study on this topic found that a majority of CLBP patients who entered the functional restoration program failed to demonstrate a normal FR pattern or normal flexion ROM. After completion of the program (with SEMGAS as a treatment component), most of them demonstrated a significantly improved FR pattern and associated ROM [29,56]. It was later demonstrated that, compared to functional restoration only, CLBP patients who participated in functional restoration with the addition of SEMGAS were significantly more successful in achieving FR after treatment completion [60]. In fact, most of these treatment patients demonstrated normalization of FR, comparable to a pain-free control group, at treatment completion. A follow-up study compared pre-treatment FR patterns and treatment responsiveness in three groups of CLBP patients: those with previous discectomies, fusions, and no previous surgeries. It was found that patients with prior surgeries initially demonstrated greater SEMG and ROM deficits, but after completing the functional restoration treatment, and participating in SEMGAS, the majority of patients in all three groups demonstrated significantly improved ROM and successfully achieved FR [61]. Two recent pilot studies, using alternative SEMGAS training methodologies than the previously cited studies, have demonstrated modest results in improving FR in groups of CLBP subjects [62,63].

## 4. Summary and Conclusions

As one can find with a “SEMG biofeedback for low back pain” literature search, the majority of clinical studies on this topic were performed in the 1980s and 1990s. Few clinical outcome studies that focus on SEMG biofeedback for CLBP have appeared in the scientific literature since that time. Perhaps these new studies on SEMGAS indicate a sign of renewed interest in SEMG biofeedback for CLBP. Some other new clinical research avenues may also show relevance for CLBP treatment. Recent studies on the use of real-time muscle monitoring of neck and shoulder muscles with a portable EMG device have been investigated with promising results. In three randomized control trials, portable EMG biofeedback was found to be effective for reducing muscular tension and associated pain and perceived disability in females with chronic neck/shoulder pain who were working in low-impact jobs (including computer work) [64,65,66]. This same technology may prove helpful for CLBP patients as well. Other new muscle-related biofeedback modalities may also be found to be useful in low back pain rehabilitation, such as real-time ultrasound imaging (RTUS) biofeedback, which provides immediate visual feedback about the shape and length of muscles as they contract and relax [3].

In conclusion, one thing is certain. Chronic pain, including CLBP, is at epidemic proportions. A new report has determined that 100 million people in the United States alone (which is almost one-third of the US population) have some form of chronic pain, and the majority of those have CLBP [67]. Thus, all therapeutic avenues, including SEMG biofeedback, should be considered within a comprehensive treatment plan for CLBP.
